# Misuse and Dependence on Non-Prescription Codeine Analgesics or Sedative H1 Antihistamines by Adults: A Cross-Sectional Investigation in France

**DOI:** 10.1371/journal.pone.0076499

**Published:** 2013-10-03

**Authors:** Anne Roussin, Annabelle Bouyssi, Lucie Pouché, Laure Pourcel, Maryse Lapeyre-Mestre

**Affiliations:** 1 Equipe de Pharmacoépidémiologie, UMR1027 INSERM- Université de Toulouse III, Toulouse, France; 2 Centre d’Evaluation et d’Information sur la Pharmacodépendance (Addictovigilance Centre), Service de Pharmacologie Clinique, Hôpitaux de Toulouse, Toulouse, France; National Taiwan University, Taiwan

## Abstract

**Background:**

Given the growing worldwide market of non-prescription drugs, monitoring their misuse in the context of self-medication represents a particular challenge in Public Health. The aim of this study was to investigate the prevalence of misuse, abuse, and dependence on non-prescription psychoactive drugs.

**Method:**

During one month, in randomly solicited community pharmacies, an anonymous questionnaire was offered to adults requesting paracetamol (control group), codeine combined with paracetamol in analgesics, or sedative H1 antihistamines. Responses about misuse (drug use not in agreement with the Patient Information Leaflet) abuse (excessive drug use having detrimental consequences), and dependence (established according to questions adapted from the Diagnostic and Statistical Manual of Mental Disorders, 4th Edition criteria) on psychoactive drugs were compared to those of the paracetamol control group.

**Results:**

295 patients (mean age 48.5 years, 68.5% of women) having used one of the studied drugs during the previous month were included. Misuse and dependence to codeine analgesics concerned 6.8% and 17.8% of the patients exposed to these drugs, respectively, (n = 118), which was significantly higher than for paracetamol. 19.5% had used codeine analgesics daily for more than six months. Headache was the most frequent reason for persistent daily use. A high prevalence of persistent daily users of sedative H1 antihistamines was also observed. Whereas these drugs are recommended only for short treatment courses of occasional insomnia, 72.2% of the participants having taken doxylamine (n = 36) were daily users, predominantly for more than six months.

**Conclusions:**

Results on misuse and dependence on non-prescription codeine analgesics suggest that chronic pain, in particular chronic cephalalgia, requires better medical care. In addition, as for hypnotics on prescription, persistent use of doxylamine for self-medication is not justified until an acceptable benefit-risk ratio for chronic sleep disturbance is shown by clinical data.

## Introduction

Drugs that can be obtained at community pharmacies without a medical prescription (non-prescription drugs) are considered to be safe enough when following the recommendations of use as presented in the Summary of Product Characteristics (SPC). Depending on the countries, patients can have free access to all or part of the non-prescription drugs in pharmacies (over-the-counter (OTC) drugs). In 2009, non-prescription drugs (not prescribed and prescribed) represented 44.9% of the drugs issued in community pharmacies in France [1]. According to Intercontinental Marketing Services (IMS) and the Association Française de l’Industrie Pharmaceutique pour une Automédication Responsable (AFIPA), in 2009 and in 2010, non-prescription drugs requested by patients without a medical prescription represented 14.1% of the total number of drug units issued in community pharmacies [2].

Among non-prescription drugs, those containing substances with a well-known potential of abuse and dependence (such as codeine) are not OTC in France, and the patients have to request them from the pharmacist. Throughout the world, problematic use of drugs containing substances with psychoactive properties used for self-medication have been recognized as an important issue in community pharmacies, particularly for opioids, anti-histamines with sedative properties, and sympathomimetics [3]–[7]. This concern was identified on the basis of the perception of the pharmacists and the knowledge of the general population about the potential problematic use of these drugs. However, there is a lack of quantitative data on misuse, abuse, and dependence on non-prescription drugs spontaneously requested by patients. Pharmacoepidemiological research on non-prescription drug use and safety are under-represented in comparison to prescription drugs. In a previous pilot study focusing on abuse of and dependence on self-medication, we have already demonstrated the feasibility to recruit patients through a regional network of community pharmacies, already involved in other studies or receiving pharmacy students for a six-month placement [8]. This study has demonstrated the feasibility and validity of a cross-sectional survey relying on an anonymous questionnaire given to patients seen in community pharmacies to investigate problematic use of psychoactive drugs used for self-medication or diversion.

The aim of the present study was to investigate the prevalence of misuse, abuse of, and dependence on codeine analgesics or on sedative H1 antihistamines for non-prescription drugs spontaneously requested by patients in community pharmacies and to identify reasons for persistent use, in comparison with non-prescription paracetamol.

## Participants and Methods

### Ethical Statement

This nationwide cross-sectional study was conducted during a one month period (from 15^th^ February to 15^th^ March 2009), relying on an anonymous questionnaire given to patients over 18 in community pharmacies. According to the French legislation on clinical research (Article 54 of the law n° 78-17 of January 6^th^ 1978 and Public Health law of August 6^th^ 2004), this study was submitted to the National Comité Consultatif sur le Traitement de l’Information en matière de Recherche dans le domaine de la Santé (CCTIRS) of the French Ministry of Research, which decided that no ethical approval was required since the patients remained anonymous. The National Council of the Pharmaceutical Order has been made aware of this study.

### Solicitation of Pharmacies

The number of pharmacies to be solicited has been calculated on the basis of the results obtained in a previous study [8]. The present survey was designed to get the highest rate of full adhesion to the protocol of the pharmacies which should accept to participate. As the pharmacies had to be solicited after a random selection based on the geographical national repartition, a low participation rate (between 5 to 10%) was anticipated. A total of 2,263 community pharmacies were solicited to participate, representing 10% of the pharmacies in each of the 22 administrative areas of the French metropolitan territory.

The pharmacies were asked to send their response by mail on their agreement to participate to the study. When they did not send back the invitation to join the study, they were asked to give their response during a telephone call from the study coordinator.

### Studied Drugs

Codeine used for analgesia is combined with paracetamol and can be purchased at community pharmacies without a medical prescription in doses up to 20 mg of codeine per pill. Thirteen analgesic formulations of drugs containing codeine can be requested without limit of duration of use. These drugs are placed behind the dispensing counter in the pharmacies, and patients must request them from a member of the pharmacy staff.

Among sedative H1 antihistamines which have been included in the study, (alimemazine, chorphenamine, dimenhydrinate, doxylamine, oxomemazine, pheniramine, and promethazine), only alimemazine, doxylamine, and promethazine are indicated in the short-term treatment of sleeping disorder in adults. According to their SPC, they must not be used for longer than five days, in the context of self-medication.

Paracetamol was included as a control. It can be obtained by requesting it from a member of the Pharmacy staff without prescription with a maximum of 8 g per box.

### Questionnaire and Data Collection

Pharmacies were randomly allocated to include patients requesting codeine combined with paracetamol, sedative H1 antihistamines, or paracetamol (with a list of all non-prescription products containing these substances). Therefore, one pharmacy had to distribute a questionnaire about only one category of the studied substances.

We asked pharmacists to offer the questionnaire to the first 12 patients requesting the studied drug during one month. Pharmacists had to explain the aim of the study to patients and to register the reasons for refusal to participate. In that case, the pharmacy staff had to offer the questionnaire to a new patient until the 12 questionnaires had been given during the study period.

The questionnaire recorded demographic information, patterns of drug use, criteria of misuse, abuse, and dependence. The misuse of paracetamol or codeine combined with paracetamol was determined when the drug was used in excessive doses (above maximal recommended doses of the majority of the SPCs of the brand drugs containing this association, i.e. 3 g/day and 120 mg/day for paracetamol and codeine phosphate, respectively), and when the use was regular (more than 10 days during the last month). For sedative H1 antihistamines, the SPC of most of the drugs specify that the treatment must not exceed five days. For this reason, misuse of the drugs containing sedative antihistamines was determined as soon as the patients declared that they used a product containing the substance for longer than five days, even when they did not use it in excessive doses.

Abuse was defined as excess use of the drug, permanently or intermittently, with detrimental consequences on the patient’s health, or social or professional life.

Substance dependence was determined according to responses to questions adapted from the Diagnostic and Statistical Manual of Mental Disorders, 4th Edition (DSM-IV criteria for problematic drug use) [9]. The patient was considered dependent when he or she met at least three criteria of physiological (tolerance and withdrawal symptoms) and/or psychological dependence (frequent use of higher doses than intended; persistent desire or unsuccessful efforts to control drug use; negative effects of the drug use on health, social, or professional life; continued drug use despite knowledge of negative effects, and a lot of time spent obtaining the drug). When the patients replied that if the drug was not available, they would not accept another drug proposed by the pharmacist and would go to another pharmacy to obtain the drug, it was considered that they spent a lot of time to obtain the drug.

The following data were also collected: purpose for taking the drug (including the use for other purpose than recommended), person who suggested their use (advice of a pharmacist, physician, or close family; after having seen an advertisement), whether the patient had informed his general practitioner (GP) about this consumption, and other drugs regularly used. Patients were free to fill out the questionnaire in the pharmacy or to take it away with prepaid envelopes to return it.

### Data Analysis

Descriptive statistics of the study population were calculated. Only the patients who had used one of the studied substances during the previous month were concerned by the questions on their patterns of use. We determined whether there were differences between each group of psychoactive substances and the control group. For continuous variables, differences were assessed using the Student’s t-test or a non-parametric test. Categorical data were described as percentages and compared using a Chi-squared test or Fisher’s exact test. Statistical analysis was conducted using SAS® 9.1 software.

## Results

### Patient Participation Rate

During the one month study period, 915 questionnaires were offered in 145 pharmacies (6.4% of the solicited pharmacies). In order to get enough questionnaires filled in by regular users of each studied substance, the number of pharmacies having to offer the questionnaire to 12 patients on codeine (n = 82) was higher than for H1 antihistamines (n = 36), and paracetamol (n = 27).

**Figure 1:**
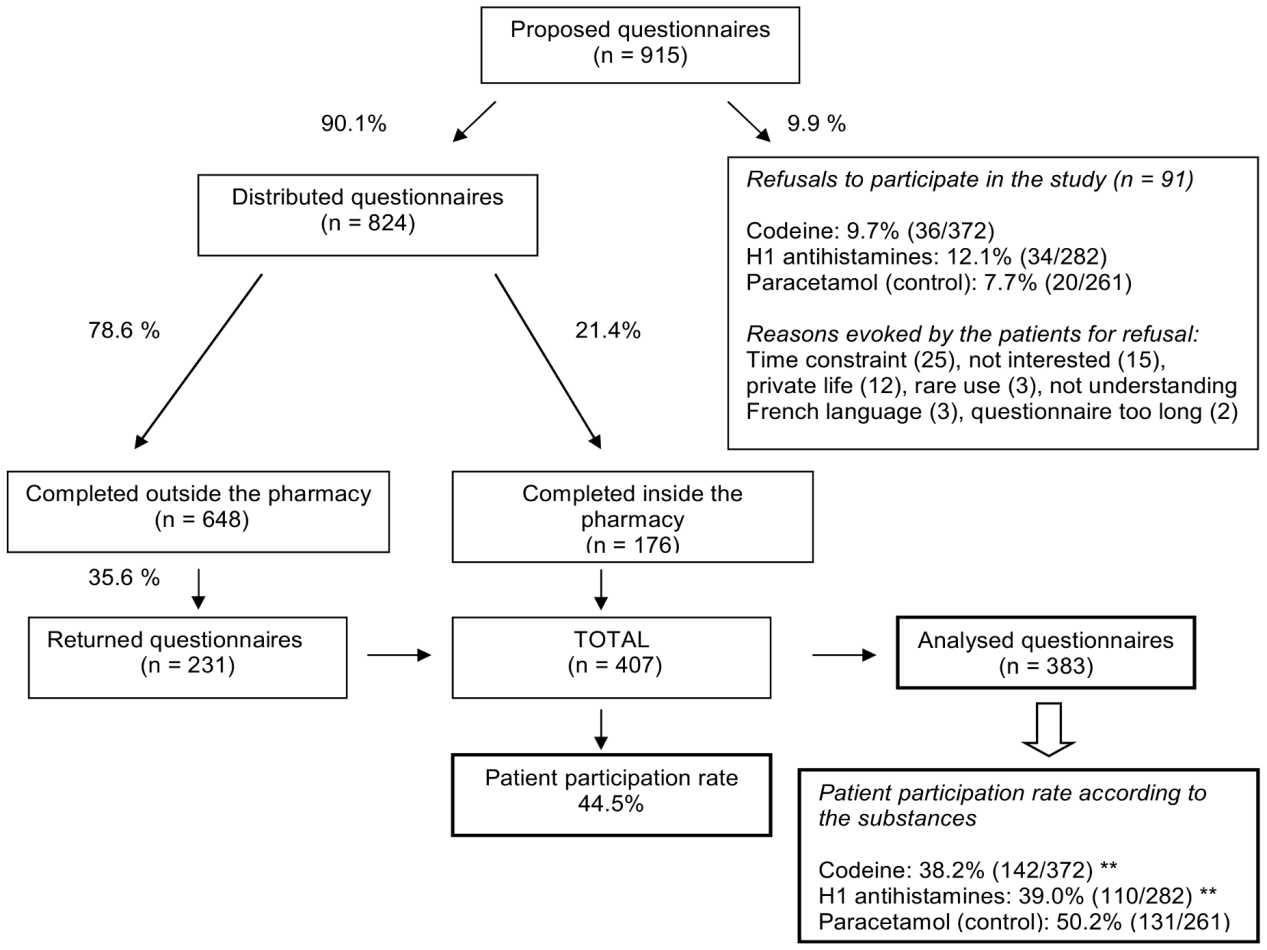
Patient participation rate. Patient participation rates were determined according to the modalities of completing the questionnaire inside or outside the pharmacy and according to the substances. (**p<0.01 vs control).

As shown in Figure 1, only 9.9% (n = 91) of the solicited patients refused to get the questionnaire. The number of refusals was not statistically different between the studied substances. The reasons given by the patients for refusal were: time constraint (25), not interested (15), indiscrete questions (12), uncommon use (3), not fluent in French language (3), too long questionnaire (2). Among the 824 distributed questionnaires, 176 (21.4%) were completed inside the pharmacy. Among the other 648 patients who accepted the questionnaire, 231 (35.6%) returned it with the prepaid envelope. A total of 407 questionnaires were received, corresponding to a patient participation rate of 44.5%. Finally, 383 questionnaires were available for analysis, with a higher participation rate in the control group with paracetamol (Figure 1).

### Patient Characteristics

The mean age was 48.0±15.9 years old and the majority (66.1%) were women. No statistically significant difference in age and sex was observed between the three studied groups of substances (Table 1).

**Table 1:** Characteristics of the patients responding to the questionnaire and of users of the studied substances in the previous month.

	Codeine	H1 antihistamines	Paracetamol (control)	Total
**Number of patients (total sample)**	142	110	131	383
Mean age (years) ± SD	46.6±13.4	48.9±15.6	48.7±18.4	48.0±15.9
Number of women	94 (66.2%)	70 (63.6%)	89 (67.9%)	253 (66.1%)
**Number of patients (users in the previous month)**	118	70	107	295
Mean age (years) ± SD	46.1±12.4	52.1±17.0	48.7±18.7	48.5±16.2
Number of women	80 (67.8%)	47 (67.4%)	75 (70.1%)	202 (68.5%)
*First use of the drug*				
On physician advice	39 (33.1%)**	16 (22.9%)***	57 (53.3%)	112 (38.0%)
On pharmacist advice	39 (33.1%)*	33 (47.1%)**	21 (19.6%)	93 (31.5%)
On close family advice	30 (25.4%)	19 (27.1%)*	16 (15.0%)	65 (22.0%)
On advertisement	3 (2.5%)	0 (0.0%)	0 (0.0%)	3 (1.0%)
Other	5 (4.2%)	1 (1.4%)	5 (4.7%)	11 (3.7%)
GP is aware of the use	79 (66.9%)	34 (48.6%)	66 (61.7%)	179 (60.7%)

* p<0.05;

** p<0.01;

*** p<0.001 (comparison to the control group).

### Patients having used the Drug in the Previous Month

A large majority of the participants (77%, n = 295) had used the drug during the previous month (Table 1). The mean age was 48.5±16.2 years old, and the majority (68.5%) were women. The patients had informed their GP about their use of the studied drug in 60.7% of cases. 38% had started use on medical advice. This rate was significantly less for codeine (33.1%) and sedative H1 antihistamines (22.9%) than for paracetamol (53.3%). In contrast, patients were significantly more numerous to have started the use of codeine (33.1%) and sedative H1 antihistamines (47.1%) than paracetamol (19.1%) on pharmaceutical advice. 22% had first purchased the drug following family advice and three percent after seeing an advertisement.

### Misuse, Abuse, Persistent use of, and Dependence on Paracetamol, Codeine Combined with Paracetamol in Analgesics and Sedative H1 Antihistamines

Frequency of misuse, abuse of, and dependence on codeine or H1 antihistamines was compared to that observed in paracetamol users (Table 2).

**Table 2:** Problematic uses of codeine (combined with paracetamol), H1 antihistamines, and paracetamol (control) among the patients having used these drugs during the previous month.

	Codeine	H1 antihistamines	Paracetamol (control)
	(N = 118)	(N = 70)	(N = 107)
Misuse	8 (6.8%)***	26 (37.1%)***	0 (0%)
Abuse	1 (0.85%)**	0 (0%)	0 (0%)
Dependence	21 (17.8%)**	1 (1.3%)	4 (3.7%)

** p<0.01;

*** p<0.001 (comparison to the control group).

### 1. Paracetamol

#### 1.1. Misuse, abuse, and persistent use of paracetamol

No patient having used paracetamol during the previous month (n = 107) declared a higher consumption than the maximal recommended dose, i.e. 4 g/day. However, the dose was unknown in 11 cases (10.3%), and four patients also used another brand drug containing paracetamol alone (two patients) or combined with codeine (two patients), without precision concerning the doses and duration of use. Whereas no misuse or abuse of paracetamol was observed, 19 patients were daily users (17.8% of the patients having used paracetamol during the previous month). Ten of them declared to have used paracetamol daily for more than six months (there were three missing data). The reasons for persistent use of paracetamol were to treat pain: 8 for musculoskeletal pain, 4 for headache pain, 1 for dental pain (no precision in 6 cases). Only one patient having a persistent use of paracetamol was qualified as dependent on this substance.

#### 1.2. Dependence on paracetamol

Among the 107 patients having used paracetamol for self-medication in the previous month, four (3.7%) were qualified as dependent on paracetamol. They were tolerant to the analgesic effects of paracetamol. They often used higher doses than intended and had a persistent desire to control drug use. All of them declared that it is the persistence of pain that led them to increase the doses they anticipated to take and also to have increased the doses of paracetamol to get the same effect as when they had begun using this drug. The description of the four cases of dependence is presented in Table 3.

**Table 3:** Cases of dependence on paracetamol purchased without prescription at the community pharmacy.

N° of case	Sex/Age (years)	Dose of paracetamol (g)	Duration of use (years)	Reason for use	Questionnaire returned in a prepaid envelope	Withdrawal symptoms (except pain)	Withdrawal causes rebound pain	Associated psychoactive drugs and analgesics	GP is awa-re of the use	Adverse effects
1	M/18	>1.5 (daily)	<0.5	toothache	No	–	yes	no	yes	no
2	F/21	missing data	10	pain	yes	–	yes	no	no	no
3	F/50	>3 (2 days/7)	5	migraine	yes	no	no	no	yes	no
4	M/25	>1 (3 days/7)	15	headache	no	no	no	ibuprofen	no	no

### 2. Codeine Combined with Paracetamol

#### 2.1. Misuse, abuse, and persistent use of codeine combined with paracetamol

Among the patients having used codeine combined with paracetamol during the previous month (n = 118), only one patient took maximal recommended doses (i.e. 4 g/day of paracetamol), but seven declared to increase sometimes or frequently the maximal recommended doses of the majority of brand drugs containing this association (i.e. 120 mg of codeine phosphate/day and 3 g of paracetamol/day). In addition, two patients reported the use of another brand drug containing codeine combined with paracetamol without specifying the dose. The description of the eight cases of misuse (6.8%) is presented in Table S1 (cases n° 1, 8, 10, 12, 13, 16, 22, and 23). Among these eight patients, six were qualified as dependent on codeine (cases n° 1, 8, 10, 12, 13, and 16). Only one patient (case n° 13) declared to use codeine in high doses for a reason other than treating pain. This 38 year-old woman had been using 200 mg of codeine phosphate and 4 g of paracetamol daily for three years, for anxiolytic effects and by habit. She declared to be dependent on codeine. Sometimes, she increases the doses, leading to an overdose of paracetamol. Another patient who misused codeine was considered as an abuser of codeine (case n° 1). This 38 year-old woman declared to have a daily consumption of codeine with intermittent use of higher doses than recommended ones for headaches. She declared suffering from deleterious consequences from her codeine consumption with a depressive mood and dependence on codeine.

Among the 118 patients, 30 (25.4%) had a daily consumption. Twenty three of them had used codeine combined with paracetamol daily for more than six months, mostly during a 2 to 5 year period (36.7%). Headache (migraine specified in nine cases) was the most frequent reason for this persistent use (for 15 patients). Musculoskeletal pain was the reason given by six patients, and the origin of pain was not specified in eight cases. One patient reported using codeine daily for three years only because of her dependence to this substance. Among the 30 persistent users of codeine combined with paracetamol, 11 were qualified as dependent on this association.

#### 2.2. Dependence on codeine combined with paracetamol

Dependence on codeine concerned 21 patients out of the 118 having used this substance for self-medication in the previous month (cases n° 1 to 21, Table S1). Ten patients presented three DSM-IV criteria of dependence, five presented four criteria, four presented five criteria, one presented six criteria, and one presented all seven criteria of dependence. Three patients declared to have a craving for codeine (cases n° 3, 10, and 13). Three patients declared the need to take codeine analgesics for reason additional to treating pain and without details (cases n° 2, 5, and 11). One person said that she used codeine for well being (case n° 10) and another one because of her dependence on codeine analgesics (case n° 13). Among the 21 cases of dependence on codeine analgesics, adverse effects were described by nine patients (Table S1). Adverse events were physical or psychological symptoms, for four and six patients, respectively. The physical symptoms declared were: constipation, nausea, vertigo, and stomach-ache. The psychological symptoms were: depressive mood, anxiety, tiredness, inattention, nervousness, and feeling sleepy.

**Figure 2:**
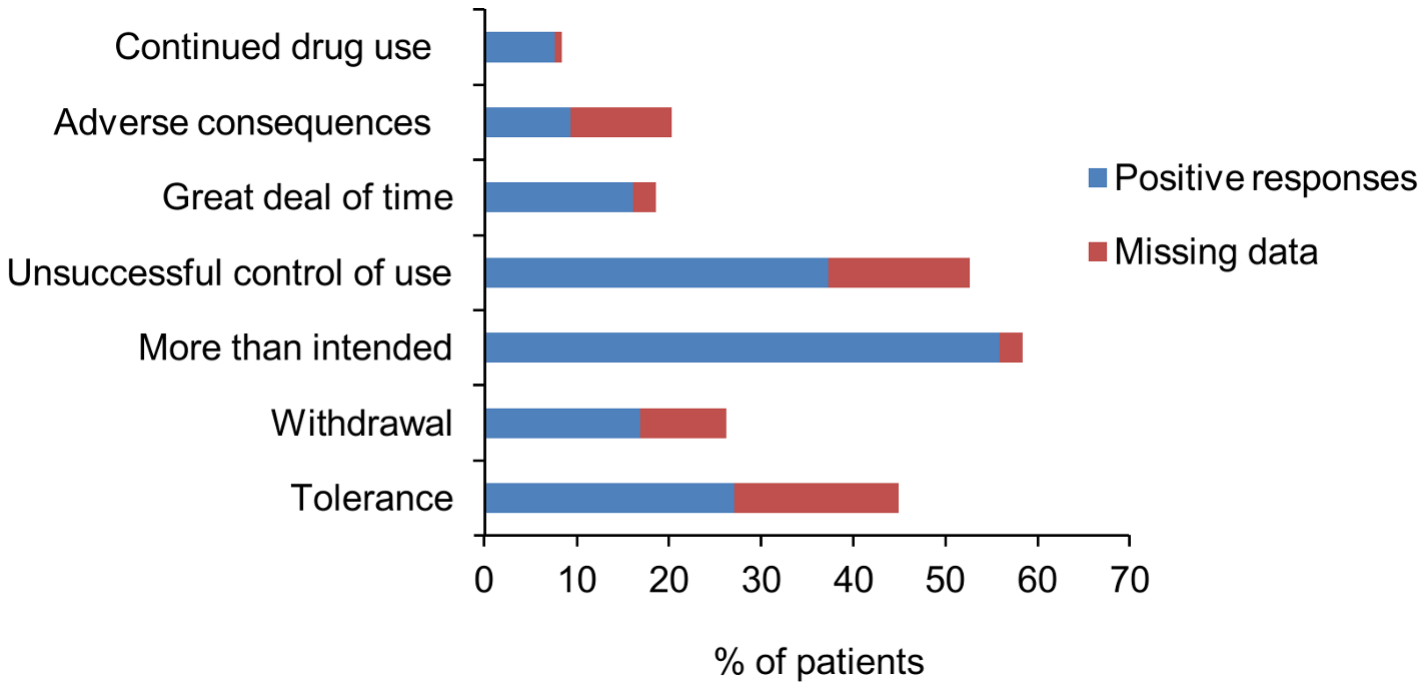
Positive responses to DSM-IV substance dependence items. Results are expressed as percentage of the patients having used non-prescription analgesic drugs containing codeine during the previous month.

A large majority of patients dependent on codeine (18 out of 21 cases) declared that persistence of pain led them to increase the doses. As shown in Figure 2, the two items of dependence of DSM-IV the most frequently retrieved were the intake of doses of codeine higher than intended (55.9%), and the persistent desire, or the unsuccessful efforts to control the consumption of codeine analgesics (37.3%).

### 3. Sedative H1 Antihistamines

#### 3.1. Misuse, abuse, and persistent use of sedative H1 antihistamines

Whereas no patient having used a sedative H1 antihistamine during the previous month (n = 70) took higher doses than recommended, 37.1% of them misused it. Concerning doxylamine, 26 patients (72.2%) out of the 36 who had used this drug during the previous month took the drug daily whereas it is recommended to stop consumption after 5 consecutive days and to obtain a physician’s advice when the insomnia persists. The description of the patients who misused doxylamine is presented in Table 4. The mean age was 59.6±14.7 years old, and the majority were women (n = 19).

**Table 4:** Cases of misuse of doxylamine (daily use for more than 5 consecutive days) purchased without prescription at the community pharmacy.

N° of case	Sex/Age (years)	Dose used daily (mg)	Duration of use (years)	Associatedpsychoactive drug	Adverse event: reboundinsomnia associated towithdrawal	Has tried todecrease dosesused but did notstop	Persistent desire orunsuccessful efforts tocontrol doxylamine use
1	F/27	30	1.5		yes	no	yes
2	F/69	30	<0.5		yes	yes	yes
3	M/59	15	18		yes	yes	yes
4	F/70	15	4	clorazepate dipotassium- acepromazine-aceprometazine	missing data	missing data	missing data
5	F/81	15	2		yes	yes	missing data
6	M/35	15	0.5		yes	yes	missing data
7	F/51	15	0.5		yes	yes	missing data
8	F/42	15	0.5		yes	yes	desires to stop but it is not a major concern
9	F/56	15	<0.5		yes	yes	yes
10	F/45	15	<0.5		no	no	yes
11	M/45	15	<0.5		yes	yes	missing data
12	F/47	15	<0.5	escitalopram and alprazolam	yes	no	yes
13	F/MV	15	<0.5		yes	yes	missing data
14	F/59	15	<0.5		no	yes	missing data
15	F/88	15	0.5	zopiclone	yes	no	no
16	M/52	7.5	5		yes	yes	yes
17	M/62	7.5	5	zopiclone, meprobamate, and valproic acid	no	yes	yes
18	M/62	7.5	2 to 5		yes	yes	missing data
19	F/82	7.5	2		yes	no	no
20	F/66	7.5	2		yes	no	missing data
21	F/59	7.5	1.5		yes	no	no
22	M/58	7.5	<0.5		yes	yes	desires to stop but it is not a major concern
23	F/72	7.5	<0.5		yes	no	yes
24	F/82	4	2 to 5		never stopped	yes	missing data
25	F/56	4	2		no	yes	missing data
26	F/65	missing data	4	valerian-passiflora-hawthorn-horehound	yes	yes	yes

In a large majority (20 out of 26) patients said that they experienced a rebound of insomnia when they did not take doxylamine or decreased the dose. No other adverse event was described by daily users except a 62 year-old man who declared to be tired upon awakening (case n° 17, Table 4). However, it was difficult to attribute this symptom to doxylamine, as this man was depressed and also used other substances which could account for this event (zopiclone, meprobamate and valproic acid). In 17 cases, patients declared that they had tried to decrease the dose but did not stop taking the drug. Ten patients had a persistent desire or made unsuccessful efforts to control doxylamine use (no precision in 11 cases). Six patients said that they would not accept another drug proposed by the pharmacist and would go to another pharmacy to obtain the drug (no response in two cases). Among the 26 patients who had taken doxylamine daily during the previous month, 16 had a daily consumption for more than six months, up to 18 years. In addition to the daily use of doxylamine, four patients also took another drug prescribed by the physician with hypnotic properties (alprazolam, meprobamate, association of clorazepate dipotassium-acepromazine and aceprometazine, or zopiclone).

#### 3.2. Dependence on H1 antihistamines

Only one patient presented three DSM-IV criteria of dependence on a drug and was a persistent user. This 55 year-old woman, declared having used doxylamine daily for sleeping for four years. She had a persistent desire to control the use of this drug. Withdrawal of doxylamine causes to her a rebound of insomnia. She was willing to spend a lot of time obtaining doxylamine. However, she did not perceive this consumption as deleterious for her health, or her social or professional life.

## Discussion

This cross-sectional study, based on the responses to an anonymous self-questionnaire offered in a randomly selected sample of community pharmacies of the French territory, has highlighted misuse of non-prescription drugs containing codeine (combined with paracetamol) or sedative H1 antihistamine (doxylamine) spontaneously requested by patients. In addition, dependence on codeine (combined with paracetamol) was also observed. Misuse and dependence on codeine analgesics concerned 6.8% and 17.8% of the patients having used this substance during the previous month, respectively, and were significantly higher than for paracetamol. For 37.1% of patients having used a sedative H1 antihistamine during the previous month, misuse of doxylamine, the most frequently reported H1 antihistamine used, concerned 72.2% of the users of this substance.

### Comparison with other Studies

The main findings obtained with drugs containing sedative H1 antihistamines concerned the high prevalence of misuse of doxylamine, i.e. a much longer use of this drug at normal dose than the recommended duration (which must not exceed five days). In our study, 72.2% of the patients who used doxylamine were daily users, and 61.5% for more than 6 months. Surprisingly, a majority of daily users of doxylamine (17 out of 26) reported informing the GP about this consumption which had started following his or her advice in 53.3% of the cases. In 4 cases out of 26, patients also used prescribed hypnotic drugs in addition to doxylamine, such as zolpidem and zopiclone.

To our knowledge, this study was the first one to investigate the reasons for the non-recommended persistent use of doxylamine. Except one patient who declared to be depressive and to use doxylamine by habit, the others reported using it for chronic sleeping problems. Data on the efficacy of persistent use of doxylamine or other first generation sedative H1 antihistamines in insomnia are scarce [10]–[12]. In a small size crossover trial design, daytime sedative effect with diphenhydramine administered twice a day was significantly higher than with placebo on day one [13]. However, tolerance to daytime sedative effect of diphenhydramine was complete after three days of administration. One randomized clinical trial of doxylamine (15 mg) versus zolpidem (10 mg), administered to 338 patients with common insomnia, has shown a similar efficacy in the two drugs with respect to sleep after two weeks of treatment; no withdrawal syndrome was observed for either drug [14]. However, data on the efficacy of longer treatment with doxylamine with respect to chronic insomnia are lacking. In our study, most persistent users of doxylamine declared to have a rebound of insomnia when they stopped or decreased the doses, and half of them reported having a persistent desire or spending a lot of time to obtain the drug. Whereas we did not find any report about the prevalence of dependence on doxylamine in the literature, cases of antihistamine abuse and dependence, especially diphenhydramine and dimenhydrinate, have been described [15]–[17]. Experimental studies performed on animals suggest the potential for abuse of and dependence on doxylamine. Doxylamine has been shown to produce a partial generalization to a 10 mg/kg cocaine training stimulus in rats [18]. Administration of doxylamine in association with diphenydramine produced complete cross-generalization with the training cue. Another reason for persistent use of doxylamine could be the low rate of adverse effects of this substance. Adverse effects of sedative antihistamines, including doxylamine, are, in particular, daytime drowsiness and altered vigilance or are associated to atropinic effects (dry mouth, constipation, and urinary retention). In our study, the population of users of doxylamine included may not have been large enough to highlight atropinic effects of doxylamine or daytime altered vigilance, even in daily users over several years. Only one of the 36 users of doxylamine during the previous month declared to be tired during awakening, but doxylamine was not the only factor potentially associated to this adverse event. A rebound of insomnia was described by 20 patients after stopping or following a lower dose of doxylamine. When observed after lowering dose of doxylamine, the rebound of sleep disturbance could correspond to a therapeutic failure. However, it was not possible to differentiate patients who experienced rebound of insomnia after withdrawal or after decreasing the doses. Therefore, it cannot be exluded that rebound of insomnia corresponds to psychological signs of withdrawal or to an anticipatory anxiety to experiment sleep disturbance when not taking the drug.

The most remarkable results obtained with codeine relate to dependence on this substance. In the literature, psychological criteria of dependence on codeine are less evoked than physiological dependence (tolerance and withdrawal symptoms) in non-cancer chronic pain patients (for a review, see for example, [19]). Surprisingly, physiological items of dependence were not the most frequently reported in our study in comparison with psychological ones. The high rate of dependence on codeine observed in this study (18% of users) seems correlated with persistence of pain, and was higher than that previously observed in one French area (7.5%) [8]. In our previous pilot study, the patient was considered dependent when three relevant behavioural criteria for established dependence concerning the harmful consequences of consumption were reported. Having taken into account the physiological criteria of dependence as well as the two other psychological criteria for dependence (often use of higher doses than intended and persistent desire or unsuccessful efforts at controling drug use) might have increased the rate of patients judged as dependent on codeine.

Whereas the potential for abuse and dependence on codeine is established, data on the prevalence and incidence of persistent use or problematic use of this weak opioid by non-painful patients or patients with non-cancer chronic pain are very few. Only few patients became persistent users (0.3%) or probable problematic users (0.08%) in a recent Norwegian study on new users of codeine [20]. However, in Norway, codeine is not available without a medical prescription form in community pharmacies. In contrast, in Australia, where codeine analgesics can be obtained OTC, warnings have been recently given on dependence on codeine [21]. Between September 2005 and September 2010, 18% of all patients referred to the Drug and Alcohol Services at the Western Hospital of Melbourne were diagnosed with opioid dependence and had medical and psychiatric problems linked to their excessive use of codeine analgesics. All had some form of chronic pain, had initiated codeine analgesic use for acute pain (e.g., headache), and all described progressive use of analgesics because of psychogenic effects (e.g., “gave me energy”, “helped me forget”) [22]. Reasons for initiation and persistence of use of codeine are very similar to those reported in our study.

### Strengths and Limitations of the Study

The study has several strengths. Firstly, this is the first study giving quantitative data on misuse, abuse of, and dependence on non-prescription drugs spontaneously requested by patients in community pharmacies. Secondly, it was based on a nation-wide randomly selected sample of pharmacies in France. A previous study in one area had already demonstrated the feasibility to recruit patients in community pharmacies to investigate problematic use of psychoactive drugs used for self-medication [8]. Thirdly, we used a control (paracetamol alone available without prescription) to get a comparative evaluation of the extent of the problem of misuse, abuse and dependence with different drugs.

The study also has several limitations. The questionnaire was given to 915 adults over 18 spontaneously requesting the studied drugs, and 44.5% of them gave the completed questionnaire either to the pharmacists or send it to the study centre. Only one third of the patients who said they would complete the questionnaire outside the pharmacy returned it with the prepaid envelope. This rate was similar to that observed in two published studies: our previous pilot study [8] and a study on pharmacovigilance of non-prescription drugs performed in the UK [23]. These studies were, to our knowledge, the only ones in which staff explained the study and asked eligible subjects to complete the questionnaire outside the pharmacy.

In our study, response rate was lower for drugs containing psychoactive substances than for the control group. Therefore, patients most concerned with a problematic use of these drugs, could have had reserves to answer the questionnaire, and the results obtained might be an underestimation, for doxylamine as well as for codeine. However, in spite of this, statistically significant higher rates of misuse or dependence have been observed with doxylamine or codeine analgesics, in comparison to the paracetamol control group.

Finally, our results cannot be extended to all the French population as children and adolescents were not included. It has been recently observed in California that most adolescent admissions in the addiction public system during 2006–2007 were for OTC drugs (32.1%), following stimulant prescription drugs (45.3%), whereas opioid prescription drugs (88.9%) were most common for adults [24]. Therefore, the number of patients with problematic uses of non-prescription drugs could have been even more elevated if young people have been included in our study.

In this study, paracetamol was used as a control. In contrast to patients dependent on codeine analgesics, the four patients presenting three DSM-IV items of dependence on paracetamol did not declare any psychoactive effects. Moreover, they did not describe any psychological symptoms when stopping use of paracetamol. Therefore, as the items of dependence for paracetamol were (i) tolerance, (ii) often used higher doses than intended and (iii) a persistent desire to control drug use, and as all the patients presented chronic pain, we conclude that it is pain itself and the anticipation of pain which lead to present items of dependence. As it has been clearly previously discussed by Lusher et al. [25], patients qualified as dependent on analgesics could present a pseudoaddiction (i.e. pain-related) or an analgesic addiction (when DSM-IV items are not related to persistence of pain but to other reasons such as well being, relaxation, euphoria,…).

### Implications for Public Health

Drugs are classified in France according to their potential to impair driving performance. A pictogram corresponding to the highest level of risk (level 3) is indicated on the package of doxylamine drugs. A recent French study has shown that level 3 prescription drugs are associated with the highest risk of traffic accidents [26]. However, no data were available for non-prescription drugs. In Australia, an association between H1 antihistamine use and motor vehicle accidents has been evidenced in professional drivers and was independent from other potentially confounding factors such as age, alcohol intake, driving exposure, and sleepiness [27]. In a randomized, placebo-controlled clinical trial in a driving simulator, it was observed that sedative antihistamines impaired simulated driving performance to a similar degree as alcohol, and, surprisingly, is not associated with sleepiness [28]. Therefore, persistent doxylamine users could be at higher risk of impaired driving performance, even in absence of drowsiness/sleepiness during daytime. Sedative H1 antihistamines have also been identified as drugs used for chemical submission (i.e. psychoactive substances administered without the knowledge of a victim in order to induce incapacitation and thus facilitate criminal actions), probably because they cause impaired vigilance and drowsiness [29].

Use of sedative H1 antihistamines was recently associated with an increased risk of mortality in comparison to non-users (HR (95% C.I.) = 4.57 (3.01 to 6.94)) in a matched cohort survival analysis performed in Pennsylvania, USA [30]. All pharmacological classes of hypnotics were associated with greater than threefold increased hazards of death, even when prescribed less than 18 pills/year; increase which was not attributable to pre-existing disease. These results could even have been underestimated as over-the-counter antihistamine sleep drugs were not included. Whereas this study did not investigate the pattern of use of sedative H1 antihistamines by the patients, it can be suspected that using higher doses than recommended ones, or having a persistent use, could worsen the associated risk of mortality.

Half of the codeine daily users reported headaches. Repeated use, without overuse, of single analgesics combined or not with opioids can lead to Medication Overuse Headache (MOH). Combination drugs containing codeine have been found to increase the risk of MOH in comparison with single analgesics [31]. Headache was also the reason for codeine use for 43.5% of the codeine-dependent patients. We did not have any information on past or present use by patients of illegal products (or legal but non-pharmaceutical products) with a potential of addiction. However, as it has been previously suggested, the need for the analgesic drug to treat headaches could result from its ability to help patients cope with life [32]. Since 2009, in England, changes in the Patient Information Leaflets and Labels on pharmaceutical products containing codeine or dihydrocodeine have been introduced to minimise the risk of overuse and addiction to these drugs. They state that the products are for short term use only, for the treatment of moderate, acute pain, and that the products can cause addiction or overuse headache if used continuously for more than three days [33]. To our knowledge, the impact of these changes in the information given to patients is still unknown.

## Conclusion

This nationwide study has shown a high prevalence of persistent users of non-prescription codeine combined with paracetamol analgesics with significant differences observed for misuse, abuse, and dependence in comparison with paracetamol alone. Among the persistent users of codeine combined with paracetamol, one third were qualified dependent and presented more frequent psychological or behavioural criteria of dependence than physiological ones. Dependence on codeine mainly seemed associated with persistence of pain. Headache was the most frequent reason for persistent daily use of codeine analgesics. These results support the fact that chronic pain requires better medical care, in particular chronic cephalalgia, which in turn will help prevent drug-related chronic headaches.

The prevalence of persistent users of doxylamine was 72.2%. A rebound of insomnia upon drug cessation, and the fact that one third of the patients declared to have a persistent desire to control doxylamine use or to make unsuccessful efforts at it suggest that it could be difficult for patients to stop taking the drug. Therefore, more data on the risk of harm associated to doxylamine or other sedative H1 antihistamines used for self-medication are required, and, as for all pharmacological classes of hypnotics, persistent use of doxylamine for chronic sleeping problem remains questionable.

Overall, the results obtained in the present study on the prevalence of problematic uses of codeine analgesics and doxylamine in non-prescription drugs in a French population sample of adults over 18 spontaneously requesting the studied drugs in pharmacy should help improve recommendations for medical care of cephalalgia and chronic sleeping problems. In addition, they also should help improve clinical management of patients dependent on these drugs.

## Supporting Information

Table S1 **Cases of misuse and dependence on drugs containing codeine combined with paracetamol purchased without prescription at the community pharmacy.**
(DOCX)
