# Supplementary material for: Misuse and Dependence on Non-Prescription Codeine Analgesics or Sedative H1 Antihistamines by Adults: A Cross-Sectional Investigation in France
Source: PLoS One. 2013 Oct 3;8(10):e76499. doi: 10.1371/journal.pone.0076499 (PMC3789666; doi:10.1371/journal.pone.0076499)
Supplement: Table S1 — Cases of misuse and dependence on drugs containing codeine combined with paracetamol purchased without prescription at the community pharmacy. (DOCX) [file pone.0076499.s001.docx]

| **No** | **Misuse** | **No of items of DSM-IV** | **Sex/Age (years)** | **Daily dose of codeine phosphate (mg)** | **Daily dose of paraceta-mol (g)** | **Duration of use (years)** | **Reason of use** | **Question-naire returned by mail** | **Withdrawal symptoms (except pain)** | **Withdrawal causes rebound pain** | **Associated psychoactive drugs, analgesics, and drugs for migraine** | **GP is aware of the use** | **Adverse effects** |
| --- | --- | --- | --- | --- | --- | --- | --- | --- | --- | --- | --- | --- | --- |
| 1 | yes | 7 | F/38 | 120 | 2.4 | 3 | migraine | yes | feeling bad | yes | naratriptan | no | depressive mood and dependence |
| 2 | no | 6 | F/47 | 40 (4 days/7) | 0.8 (4 days/7) | 3 | migraine | yes | vomiting and cardiac palpitations | yes | frovatriptan | yes | anxiety and tiredness |
| 3 | no | 5 | F/51 | 80 (3 days/7) | 1.6 (3 days/7) | 5 | articular pain | no | nausea | yes | aripiprazole, paroxetine, prazepam, alimemazine, and alcohol | no |  |
| 4 | no | 5 | F/56 | 80 | 1.6 | 2 | pain | no | no | yes | no | no | no |
| 5 | no | 5 | M/42 | 80 | 1.6 | 2 | migraine | yes | no | yes | paracetamol, ibuprofen, and zolmitriptan | yes | no |
| 6 | no | 5 | M/36 | 80 | 1.6 | 0.5 | headache | no | no | yes | _ | no | inattention |
| 7 | no | 4 | M/64 | 120 | 2.4 | 10 | back pain | yes | anxiety of rebound pain | _ | _ | yes | constipation |
| 8 | yes | 4 | F/42 | 160 | 3.2 | 10 | pain | no | feeling bad | yes | _ | yes |  |
| 9 | no | 4 | F/26 | 80 | 1.6 | <0.5 | migraine and cervicalgia | yes | aches | yes | no | no | tiredness and nervousness |
| 10 | yes | 4 | F/47 | 160 | 3.2 | 1 | backpain and well being | yes | nervousness | yes | no | no | constipation and vertigo |
| 11 | no | 4 | M/39 | 120 | 2.4 | 2.5 | traumatism | no | celiac plexus pain | yes | no | yes | stomach-ache |
| 12 | yes | 3 | M/55 | 120 | 2.4 | 3 | cervicalgia | no | _ | yes | codeine-paracetamol, chlorazepate dipotassium,acepromazine,aceprométazine | yes | no |
| 13 | yes | 3 | F/38 | 200 | 4 | 3 | dependence (anxiety and habit) | yes | feeling bad and sweating | _ | fluoxetine and doxylamine | yes | no |
| 14 | no | 3 | F/34 | 40 (3 days/7) | 0.8 (3 days/7) | 3 | articular pain | yes | no | yes | fluoxetine and valproic acid | yes | feeling sleepy |
| 15 | no | 3 | M/73 | 20 (2 days/7) | 0.4 (2 days/7) | 10 | pain | no | no | no | no | yes | no |
| 16 | yes | 3 | M/42 | 120 | 2.4 | >1 | migraine | yes | no | yes | codeine-paracetamol, aspirin, and ibuprofen (not prescribed) | yes | no |
| 17 | no | 3 | F/47 | 40 | 1 | 3 | headache | yes | no | no | naratriptan | yes | no |
| 18 | no | 3 | F/21 | 120 (1 day/7) | 2.4 (1 day/7) | 3 | headache | no | nausea | yes | paracetamol and aspirin (not prescribed) | no |  |
| 19 | no | 3 | F/63 | 40 (2 days/7) | 0.8 (2 days/7) | 5 | headache | yes | no | yes | meprobamate | yes | no |
| 20 | no | 3 | F/25 | 40 (4 days/7) | 0.8 (4 days/7) | 5 | migraine | no | no | no | codeine-paracetamol and tramadol-paracetamol | yes | nausea, constipation, feeling sleepy |
| 21 | no | 3 | M/39 | 40 | 0.8 | 4 | abdominal pain | no | no | yes | paracetamol | yes | no |
| 22 | yes | 1 | F/44 | 180 (6 days/7) | 3.6 (6 days/7) | 20 | headache | no | no | no | no | yes | no |
| 23 | yes | 1 | F/32 | 120 (6 days/7) | 2.4 (6 days/7) | <0.5 | dental pain | no | no | no | no | yes | no |
